# Complete genome assemblies and antibiograms of 22 *Staphylococcus capitis* isolates

**DOI:** 10.1186/s12863-025-01303-8

**Published:** 2025-02-15

**Authors:** Yu Wan, Rachel Pike, Alessandra Harley, Zaynab Mumin, Isabelle Potterill, Danièle Meunier, Mark Ganner, Maria Getino, Juliana Coelho, Elita Jauneikaite, Kartyk Moganeradj, Colin S. Brown, Alison H. Holmes, Alicia Demirjian, Katie L. Hopkins, Bruno Pichon

**Affiliations:** 1 HCAI, Fungal, AMR, AMU and Sepsis Division, UK Health Security Agency, London, United Kingdom; 2 NIHR Health Protection Research Unit in Healthcare Associated Infections and Antimicrobial Resistance, Department of Infectious Disease, Imperial College London, London, United Kingdom; 3 David Price Evans Global Health and Infectious Diseases Research Group, Institute of Systems, Molecular and Integrative Biology, University of Liverpool, Liverpool, United Kingdom; 4 Public Health Microbiology Reference Services, Specialised Microbiology & Laboratories, UK Health Security Agency, London, United Kingdom; 5 Department of Infectious Disease Epidemiology, School of Public Health, Imperial College London, London, United Kingdom; 6 Centre for Antimicrobial Optimisation, Hammersmith Hospital, Imperial College London, London, United Kingdom; 7 Paediatric Infectious Diseases and Immunology, Evelina London Children’s Hospital, London, United Kingdom; 8 Faculty of Life Sciences & Medicine, King’s College London, London, United Kingdom

**Keywords:** *Staphylococcus capitis*, Reference genomes, Hybrid genome assembly, Antimicrobial susceptibility, Antimicrobial resistance, Antibiograms, Bioresource, Genomics, NRCS-A clone, Nanopore MinION sequencing

## Abstract

**Objective:**

*Staphylococcus capitis* is part of the human microbiome and an opportunistic pathogen known to cause catheter-associated bacteraemia, prosthetic joint infections, skin and wound infections, among others. Detection of *S. capitis* in normally sterile body sites saw an increase over the last decade in England, where a multidrug-resistant clone, NRCS-A, was widely identified in blood samples from infants in neonatal intensive care units. To address a lack of complete genomes and antibiograms of *S. capitis* in public databases, we performed long- and short-read whole-genome sequencing, hybrid genome assembly, and antimicrobial susceptibility testing of 22 diverse isolates.

**Data description:**

We present complete genome assemblies of two *S. capitis* type strains (subspecies *capitis*: DSM 20326; subspecies *urealyticus*: DSM 6717) and 20 clinical isolates (NRCS-A: 10) from England. Each genome is accompanied by minimum inhibitory concentrations of 13 antimicrobials including vancomycin, teicoplanin, daptomycin, linezolid, and clindamycin. These 22 genomes were 2.4–2.7 Mbp in length and had a GC content of 33%. Plasmids were identified in 20 isolates. Resistance to teicoplanin, daptomycin, gentamicin, fusidic acid, rifampicin, ciprofloxacin, clindamycin, and erythromycin was seen in 1–10 isolates. Our data are a resource for future studies on genomics, evolution, and antimicrobial resistance of *S. capitis*.

**Supplementary Information:**

The online version contains supplementary material available at 10.1186/s12863-025-01303-8.

## Objective

*Staphylococcus capitis*, consisting of two subspecies *capitis* and *urealyticus* [1, 2], is a coagulase-negative opportunistic pathogen commonly causing late-onset sepsis (LOS) in very-low-birthweight infants in neonatal intensive care units (NICUs) and various infections in adults, such as prosthetic joint infections [3, 4]. A multidrug-resistant clone of *S. capitis* subsp. *urealyticus*, NRCS-A, has emerged as a global concern in neonatal health for its dominance in LOS, reduced susceptibility to vancomycin, enhanced biofilm-forming ability, increased disinfectant and desiccation tolerance, association with neonatal incubators, and persistence in NICUs [5–7]. Between 2020 and 2021, the UK Health Security Agency (UKHSA) convened a nationwide investigation into increased reporting of neonatal *S. capitis* bacteraemia in England [8] and requested voluntary referral of clinical isolates from diagnostic laboratories for whole-genome sequencing (WGS). Genomic epidemiological analysis of the WGS data revealed widespread presence of the NRCS-A clone in neonatal units across the country, highlighting the need to further understand genomics, antimicrobial resistance (AMR), and niche adaptation of this clone in comparison with other *S. capitis* subpopulations [9].

In this report, we describe complete genome assemblies and accompanying antibiograms of 22 *S. capitis* isolates consisting of 20 English clinical isolates and type strains of the two subspecies (*capitis*: DSM 20326; *urealyticus*: DSM 6717). The clinical isolates were selected from UKHSA’s culture collection to represent the main *S. capitis* subpopulations previously identified (Figure S1) [9], with 19 of these isolates recovered from normally sterile sites in patients across England. Readers are referred to Table S1 for source information of isolates. Altogether, 10 NRCS-A isolates and 12 non-NRCS-A isolates were sequenced. Our work addresses the lack of finished-grade *S. capitis* reference genomes and antibiograms in the National Center for Biotechnology Information (NCBI) databases, providing a bioresource for future studies on the genomics, AMR, molecular epidemiology, and evolution of *S. capitis*.

## Data description

Each isolate was incubated on Columbia agar with horse blood (PB0122A, Thermo Scientific, UK) at 37 °C overnight for DNA extraction and antimicrobial susceptibility testing in 2021 (Batch 1, 19 isolates) or 2023 (Batch 2, three isolates) (Table S2). For Batch 1, genomic DNA of each isolate was extracted using GeneJET Genomic DNA Purification Kits (Thermo Scientific) and aliquoted. Long-read WGS was conducted with Oxford Nanopore Technologies (ONT, UK) MinION R9.4.1 flow-cells (FLO-MIN106D) and Rapid Barcoding Kits (SQK-RBK004). High-accuracy basecalling was performed with guppy (ONT). Short-read sequencing was performed on Illumina HiSeq 2500 systems at UKHSA following an in-house 2 × 101 bp protocol. For Batch 2, genomic DNA was extracted using a Wizard Genomic DNA Purification Kit (Promega, USA) and aliquoted. Long-read WGS was conducted with an ONT MinION R10.4.1 flow-cell (FLO-MIN114) and Rapid Barcoding Kit V14 (SQK-RBK114.24). Super-accuracy basecalling was performed using guppy. Short-read sequencing was performed under a 2 × 251 bp layout on Illumina NovaSeq 6000 systems at MicrobesNG (UK). Susceptibility of isolates to 13 antimicrobials was determined by the UKHSA Antimicrobial Resistance and Healthcare Associated Infections Reference Unit with gradient strips (Liofilchem, Italy, for Batch 1) or broth microdilution (EUSTAPF, Thermo Scientific, for Batch 2) following European Committee on Antimicrobial Susceptibility Testing (EUCAST) breakpoints v13.1. Inducible clindamycin resistance was sought by testing for the antagonism between erythromycin and clindamycin (D-test).

ONT reads were trimmed and filtered with fastp and nanoq [10, 11], respectively, for quality control. Illumina reads were trimmed and filtered with fastp. Hybrid assembly was performed for each genome using an ONT-reads-first strategy with Flye, Raven, and miniasm-minipolish, as implemented in Trycycler [12–15], when quality-processed ONT reads had an estimated depth of ≥ 60 folds and the assemblers produced consistent results; otherwise, Raven was used. When a genome could not be fully assembled from ONT reads, an Illumina-reads-first strategy was applied using Unicycler [16, 17]. All assemblies were polished with ONT reads using medaka (https://github.com/nanoporetech/medaka) followed by Illumina-read polishing using Polypolish and POLCA [18, 19]. Polished assemblies were then reoriented to start from *dnaA* (chromosomes) or *repA* (plasmids) genes using dnaapler [20]. Reoriented assemblies were polished with Illumina reads using Polypolish, assessed using Quast and CheckM2 [21, 22], and annotated with the NCBI Prokaryotic Genome Annotation Pipeline [23].

Twenty-two complete genome assemblies were generated, with the same GC content of 33% and lengths of 2.4–2.7 Mbp (Table S2). One to four plasmids (2.3–69 kbp) were identified in 20 isolates (NRCS-A: 9; non-NRCS-A: 11). The NRCS-A clone exhibited reduced susceptibility to teicoplanin, daptomycin, and gentamicin (Table S3). Resistance to fusidic acid (MICs: 16–32 mg/L), erythromycin (MIC > 256 mg/L), and clindamycin (MIC > 256 mg/L or inducible) was seen in 9/22 (41%), 7/22 (32%), and 5/22 (23%) of isolates, respectively, with no significant frequency difference between NRCS-A and non-NRCS-A isolates (*p*-value = 1, Fisher’s exact test). One NRCS-A isolate exhibited rifampicin resistance (MIC > 32 mg/L). All isolates were susceptible to vancomycin, linezolid, and quinupristin-dalfopristin, and seven non-NRCS-A isolates, including the two type strains, were susceptible to all 11 antimicrobials having EUCAST breakpoints.

## Limitations

This dataset is limited to a small sample size (*n* = 22), which does not capture all major phenotypic and genetic variations in *S. capitis*. The isolates are limited to England and dominated by invasive isolates (*n* = 19) recovered from normally sterile body sites of humans — eight (42%) invasive isolates were collected from infants of ≤ 90 days of age, one (5%) from an infant between > 90 days and < 1 year of age, two (10%) from children between six and 11 years of age, and eight from adults (≥ 18 years of age). Moreover, antimicrobial susceptibility of all isolates in Batch 1 (*n* = 19) was not determined using the gold-standard method, broth microdilution, owing to technical unavailability. Future work needs to elucidate mechanisms of AMR [24] and include a wider range of isolates, such as those recovered from carriage screening, environments, animals, and other health-related samples from non-clinical settings.

**Table 1:** Overview of datafiles/datasets

Label	Name of data file/data set	File types (file extension)	Data repository and identifier (DOI or accession number)
Datafile 1	Figure S1, Genetic relatedness between the 22 selected isolates within the context of 838 *S. capitis* isolates previously analysed	Portable Document Format file (.pdf)	Figshare, 10.6084/m9.figshare.26048464 [25]
Datafile 2	Table S1, sources and data accessions of the 22 *S. capitis* isolates	MS Excel file (.xlsx)	Figshare, 10.6084/m9.figshare.26049013 [26]
Datafile 3	Table S2, methods and summary statistics of 22 hybrid genome assemblies	MS Excel file (.xlsx)	Figshare, 10.6084/m9.figshare.26049070 [27]
Datafile 4	Table S3, antibiograms of the 22 *S. capitis* isolates	MS Excel file (.xlsx)	Figshare, 10.6084/m9.figshare.26049094 [28]
Dataset 1	Illumina and ONT whole-genome sequencing reads of the 22 *S. capitis* isolates	FASTQ (.fastq)	Sequence Read Archive, http://identifiers.org/insdc.sra: SRP483965 [29]
Dataset 2	Genome assembly of isolate DSM20326	FASTA (.fna)	Genome Assembly, http://identifiers.org/insdc.gca: GCA_040739495 [30]
Dataset 3	Genome assembly of isolate DSM6717	FASTA (.fna)	Genome Assembly, http://identifiers.org/insdc.gca: GCA_040739365 [31]
Dataset 4	Genome assembly of isolate Sc1191092	FASTA (.fna)	Genome Assembly, http://identifiers.org/insdc.gca: GCA_040739255 [32]
Dataset 5	Genome assembly of isolate Sc1191106	FASTA (.fna)	Genome Assembly, http://identifiers.org/insdc.gca: GCA_040739205 [33]
Dataset 6	Genome assembly of isolate Sc1365665	FASTA (.fna)	Genome Assembly, http://identifiers.org/insdc.gca: GCA_040739095 [34]
Dataset 7	Genome assembly of isolate Sc1365666	FASTA (.fna)	Genome Assembly, http://identifiers.org/insdc.gca: GCA_040738865 [35]
Dataset 8	Genome assembly of isolate Sc1365669	FASTA (.fna)	Genome Assembly, http://identifiers.org/insdc.gca: GCA_040738415 [36]
Dataset 9	Genome assembly of isolate Sc1365670	FASTA (.fna)	Genome Assembly, http://identifiers.org/insdc.gca: GCA_040737765 [37]
Dataset 10	Genome assembly of isolate Sc1365673	FASTA (.fna)	Genome Assembly, http://identifiers.org/insdc.gca: GCA_040737135 [38]
Dataset 11	Genome assembly of isolate Sc1365674	FASTA (.fna)	Genome Assembly, http://identifiers.org/insdc.gca: GCA_040737085 [39]
Dataset 12	Genome assembly of isolate Sc1365682	FASTA (.fna)	Genome Assembly, http://identifiers.org/insdc.gca: GCA_040736935 [40]
Dataset 13	Genome assembly of isolate Sc1365688	FASTA (.fna)	Genome Assembly, http://identifiers.org/insdc.gca: GCA_040736845 [41]
Dataset 14	Genome assembly of isolate Sc1365695	FASTA (.fna)	Genome Assembly, http://identifiers.org/insdc.gca: GCA_040736815 [42]
Dataset 15	Genome assembly of isolate Sc1365701	FASTA (.fna)	Genome Assembly, http://identifiers.org/insdc.gca: GCA_040736805 [43]
Dataset 16	Genome assembly of isolate Sc1365706	FASTA (.fna)	Genome Assembly, http://identifiers.org/insdc.gca: GCA_040736795 [44]
Dataset 17	Genome assembly of isolate Sc1365750	FASTA (.fna)	Genome Assembly, http://identifiers.org/insdc.gca: GCA_040736725 [45]
Dataset 18	Genome assembly of isolate Sc1365753	FASTA (.fna)	Genome Assembly, http://identifiers.org/insdc.gca: GCA_040736715 [46]
Dataset 19	Genome assembly of isolate Sc1365754	FASTA (.fna)	Genome Assembly, http://identifiers.org/insdc.gca: GCA_040736665 [47]
Dataset 20	Genome assembly of isolate Sc1516939	FASTA (.fna)	Genome Assembly, http://identifiers.org/insdc.gca: GCA_040736635 [48]
Dataset 21	Genome assembly of isolate Sc1516941	FASTA (.fna)	Genome Assembly, http://identifiers.org/insdc.gca: GCA_040736585 [49]
Dataset 22	Genome assembly of isolate Sc1516943	FASTA (.fna)	Genome Assembly, http://identifiers.org/insdc.gca: GCA_040736555 [50]
Dataset 23	Genome assembly of isolate Sc1516945	FASTA (.fna)	Genome Assembly, http://identifiers.org/insdc.gca: GCA_040736045 [51]

## Electronic supplementary material

Below is the link to the electronic supplementary material.

Supplementary Material 1

Supplementary Material 2

Supplementary Material 3

Supplementary Material 4

## Data Availability

Data generated in this study are listed in Table 1. UK clinical isolates are available at the UKHSA *Staphylococcus* and *Streptococcus* Reference Service. Type strains DSM 20326 and DSM 6717 are available in the DSMZ-German Collection of Microorganisms and Cell Cultures GmbH, Leibniz Institute (https://www.dsmz.de).

## Abbreviations

AMR: Antimicrobial resistance
DHSC: Department of Health and Social Care
DSM: Deutsche Sammlung von Mikroorganismen
EUCAST: European Committee on Antimicrobial Susceptibility Testing
LOS: Late-onset sepsis
NCBI: National Center for Biotechnology Information
NICU: Neonatal intensive care unit
NIHR: National Institute for Health and Care Research
ONT: Oxford Nanopore Technologies
WGS: Whole-genome sequencing
UKHSA: UK Health Security Agency
