# Supplementary material for: Complete genome assemblies and antibiograms of 22 Staphylococcus capitis isolates
Source: BMC Genom Data. 2025 Feb 15;26:12. doi: 10.1186/s12863-025-01303-8 (PMC11830182; doi:10.1186/s12863-025-01303-8)
Supplement: Supplementary file 4 — Supplementary Material 4 [file 12863_2025_1303_MOESM4_ESM.pdf]

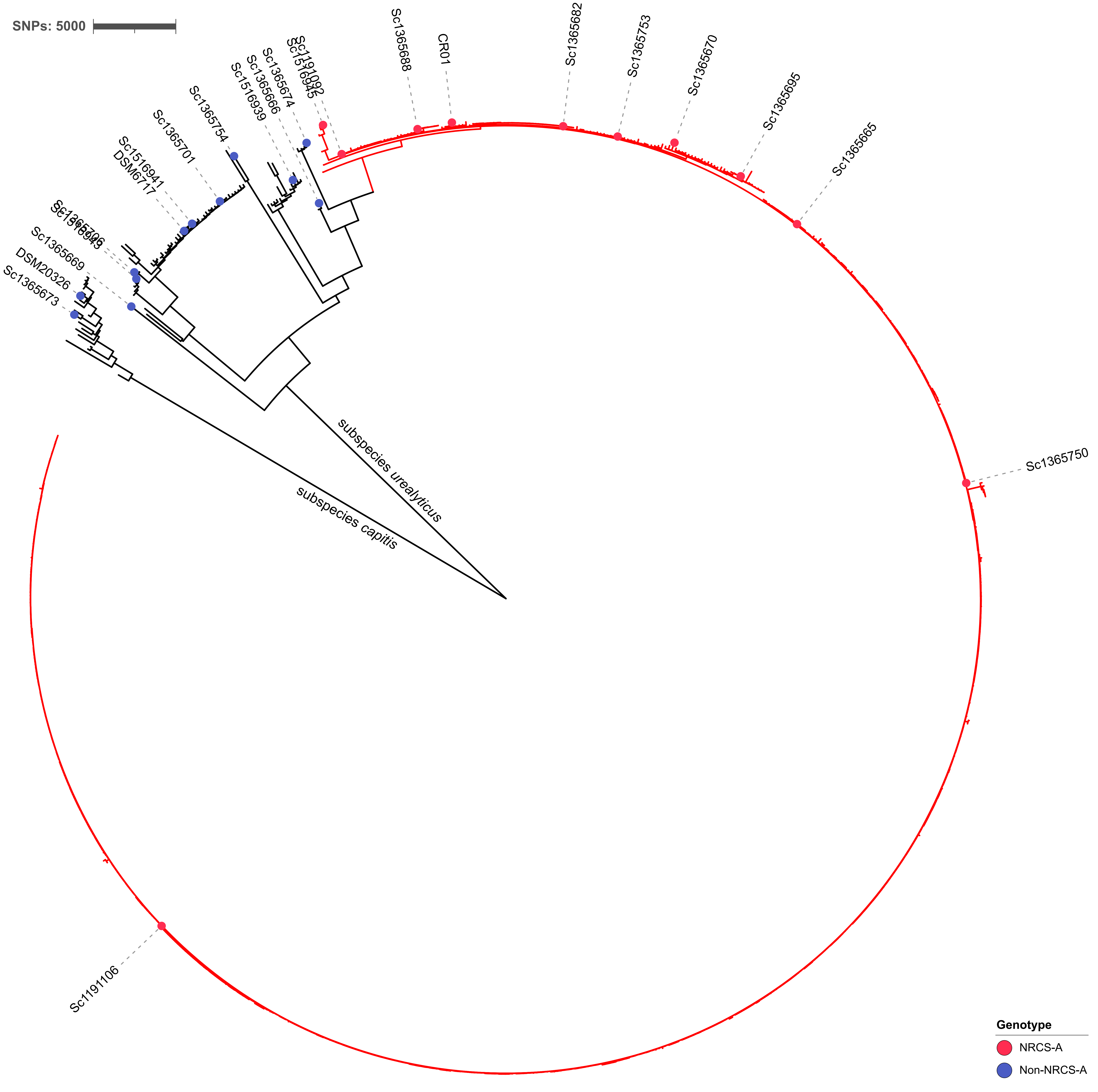

Figure S1. Genetic relatedness between the 22 selected isolates within the context of 838 *S. capitis* isolates previously analysed (Wan *et al*., *Journal of Infection*, 2023; https://doi.org/10.1016/j.jinf.2023.06.020), as illustrated in a neighbour-joining tree based on core-gene single-nucleotide polymorphisms (SNPs) identified using ggCaller v1.3.4 (https://github.com/bacpop/ggCaller). The tree is midpoint-rooted and visualised using iTOL (https://itol.embl.de). Tip circles and dashed lines indicate positions of the 22 isolates in the tree.
